# Fishing for food: Values and benefits associated with coastal infrastructure

**DOI:** 10.1371/journal.pone.0249725

**Published:** 2021-04-15

**Authors:** Cassandra M. Nieman, Alexie N. Rudman, Margaret L. Chory, Grant D. Murray, Luke Fairbanks, Lisa M. Campbell

**Affiliations:** 1 Duke University Marine Lab, Nicholas School of the Environment, Duke University, Beaufort, North Carolina, United States of America; 2 Oak Ridge Institute for Science and Education, Environmental Protection Agency, Washington, DC, United States of America; 3 Division of Coastal Sciences, School of Ocean Science and Engineering, The University of Southern Mississippi, Ocean Springs, Mississippi, United States of America; University of New Haven, UNITED STATES

## Abstract

While there is substantial literature about the socio-cultural characteristics and values associated with recreational and commercial fisheries in the U.S., studies directed at those who ‘fish for food’—those who depend on consuming their catch to various degrees—are relatively sparse. Using qualitative data collected through 80 semi-structured interviews with fishers in the summer and fall of 2018 in Carteret County, North Carolina, this study aims to better understand the group of recreational fishers who consume their catch by describing social and cultural dimensions and values associated with fishing for food, examining the role of infrastructure in facilitating access to benefits associated with this activity, and considering how knowledge of existing licensing regulations surrounding subsistence license waivers affect this fishing community. Interviews conducted at free public fishing structures in the region revealed that fishers derive a variety of values and benefits from fishing at these sites, including access to recreation, nutrition, a social community, and mental health benefits, which were found to be negatively impacted by Hurricane Florence in September 2018. We also found an informal economy of sharing catch on- and off-site that extends the reach and benefits facilitated by public infrastructure to people beyond those using it directly. Overall, we call for conceptualizations of ‘fishing for food’ that include aspects that go beyond traditional definitions of ‘subsistence’ or ‘recreational’ fishing such as food security, access, and less obvious social and cultural motivations behind the activity. These findings are a compelling rationalization for the creation and maintenance of formal and informal fishing places locally and, by extension, in other coastal areas, given the array of benefits provided by access to these types of locations.

## 1. Introduction

Recreational and subsistence fishing have long been important staples of coastal economies and communities in the United States and elsewhere. Traditionally associated with the notion of fishing for sport or enjoyment, the Food and Agriculture Organization characterizes recreational fishing as the “fishing of aquatic animals (mainly fish) that do not constitute the individual’s primary resource to meet basic nutritional needs and are not generally sold or otherwise traded on export, domestic, or black markets” [1]. On the other hand, subsistence fishing, which tends to be less visible in nature, is a term that “implies bare existence” and is often conceptualized as a non-market survival strategy [2]. In the United States, subsistence fishing has primarily been studied in the context of indigenous communities. Along with commercial fishing, recreational and subsistence fishing have been used to categorize different types of fishing globally. Complementing the body of literature that explores the motivations, values, and management considerations associated with these types of fishing, this article focuses on the individuals ‘fishing for food’ to suggest that the boundaries of these definitions have led to some inadequacy in accounting for the different types of values and benefits associated with some types of fishing.

Prominent definitions of recreational fishing can overlook important values associated with the activity. Cooke et al., for example, criticize the Food and Agriculture Organization’s definition of recreational fishing for failing to recognize the possible value and satisfaction of harvesting and consuming catch [3]. They also note the misleading implications associated with the term “recreational,” which suggests that it occurs during non-work hours or leisure time [3]. The Cooke et al. study is not alone in suggesting that traditional conceptions of recreational fishing overlook the value of consuming catch. In a study on self-identified recreational anglers in Northeastern U.S. coastal counties, 28% cited fishing for reasons other than purely for recreation, which included fishing for food and supplementary income [4]. In that study, 17.8% of anglers stated they at least partially relied on “self- caught marine resources as a cost-saving food source” and 2.4% indicated they sold their catch to supplement their income [4]. Similarly, in a different study situated in Tyrrell County, North Carolina, researchers found that a significant portion of the county’s low-income residents depend on recreationally caught fish, illustrating the role of recreational fishing as a means to access food [5].

The definition of subsistence fishing is equally limited. With an emphasis on fishing for survival, the term “subsistence fishing” obscures the diversity of values associated with the activity, which can range from relaxation and enjoyment, to spending time with one’s family, and carrying on important cultural traditions [6]. Moreover, it is a label that does not resonate with all fishers that consume their catch. In a study on fishing for food in urban Connecticut, for example, nearly half of the fishers surveyed were aware of the term ‘subsistence,’ but did not consider themselves to fall into this category, despite 43% of respondents citing fishing for food as an important motivation for fishing [6].

This study aims to better describe the group of recreational fishers who ‘fish for food’- those who depend on consuming their catch to various degrees- and the range of values these fishers associate with fishing for food that have been obscured in traditional definitions of both recreational and subsistence fishing. While relatively sparse, the existing literature on ‘fishing for food’ suggests aspects that go beyond traditional definitions of ‘subsistence’ or ‘recreational’ fishing, to include things like food security and access as well as less obvious social and cultural motivations behind the activity. It is a practice that has socio-cultural characteristics, a range of values associated with it, and merits management considerations that are distinct from (though related to) those traditionally associated with recreational and subsistence fishing.

Though food security is a critical component of fishing for food, it is clear that personal consumption is not the only motivation for the activity. Fishing for food can also play an important role in supporting social networks and informal economies as individuals share their catch with friends, neighbors, family, and others. For example, in a study conducted by Pulford, Polidoro, and Nation characterizing the relationship between water quality, recreational fishing, and human health in Arizona’s low and high income neighborhoods, the authors found that the majority of fishers surveyed were consuming their catch and sharing it with family, friends, and neighbors [7]. Some fishers who did not eat the fish they caught cited giving away their fish to others [7]. Another study in urban Connecticut similarly explored the informal economy of sharing and trading fish, and found that a high percentage (68%) of fishers that consumed their catch shared it with others, including friends and family [6]. One of the key objectives of this study was therefore to complement this small yet growing body of research which suggests that fishing for food serves social and economic functions beyond subsistence consumption.

While access to fishing areas is intuitively important, few studies, particularly in the U.S., have focused specifically on the values and benefits that access to physical fishing infrastructure provides to those fishing for food. Rather than focusing on the everyday value of fishing infrastructure, most studies examining the value of coastal infrastructure describe economic and social impacts of natural disasters on commercial fishing infrastructure [8] or individual property like fishing equipment and gear [9, 10]. One of the few accounts of the values fishers place on coastal fishing infrastructure, and benefits provided by access to it, surrounds the privatization of the previously publicly accessible port areas and piers in Durban, South Africa. The politically and economically driven securitization of Durban’s port with fences, gates, and security checkpoints barring access to water has excluded a well-established subsistence fishing community from this public space and infrastructure [11, 12]. Descriptions of the historical and current value of the public port area, including access to its piers, reveal that the subsistence fishing community values access for reasons of trade and family tradition, social support systems between fishers, their heritage and lifestyle, personal and community identity, as well as economic benefits [11]. With the exception of this Durban case study, research focused on the value of access to infrastructure for people fishing for food remains sparse in the literature.

This study attempts to help fill that gap, by exploring how in Carteret County, access to infrastructure could facilitate the myriad values and benefits that fishers gain from fishing for food. In particular, we set out to document which physical characteristics make different types of infrastructure desirable, the values that fishers associate with that infrastructure, and how physical access to infrastructure facilitates nutritional, social, recreational, cultural, and mental health benefits associated with fishing for food. Fishing for food can happen from a wide variety of platforms—from the natural shoreline, from built infrastructure (public and private), from privately owned vessels, or from charter and head boats. We chose to focus specifically on public infrastructure to make this project logistically feasible and to focus on activities where the costs to participate were low. In this study, public infrastructure refers to both formal and informal infrastructure from which people can fish for free. Formal infrastructure refers to locations and structures built for the purpose of fishing and includes public boat ramps or piers. Informal infrastructure refers to structures built for other purposes but used for fishing, like bridges.

A third major motivation for this study was to explore how the manner in which fishing is managed, including access to licensing information, can serve to limit or enable access to fishing. For example, a study in Tyrrell County, North Carolina, demonstrated that the cost of a fishing license and the lack of knowledge of “subsistence fishing waivers” among 50% of study respondents impeded fishers’ abilities to access fish [5], so we chose to examine fishers’ access to licensing information. We were interested in fishers’ knowledge of licensing requirements because of the potential for unlicensed individuals to be overlooked in official recreational fishing records and, therefore, in management decisions. We also chose to explore whether improving awareness of certain licensing regimes might serve to ensure access to fishing, potentially improving food security.

With these guiding considerations, this study focuses on Carteret County, North Carolina, and has the following specific objectives 1) to describe social and cultural dimensions and values associated with fishing for food, 2) to explore the role of infrastructure in facilitating access to benefits and values associated with this activity, and 3) to consider how knowledge of existing licensing regulations surrounding subsistence license waivers affect this fishing community.

## 2. Methodology & study design

**Fig 1:**
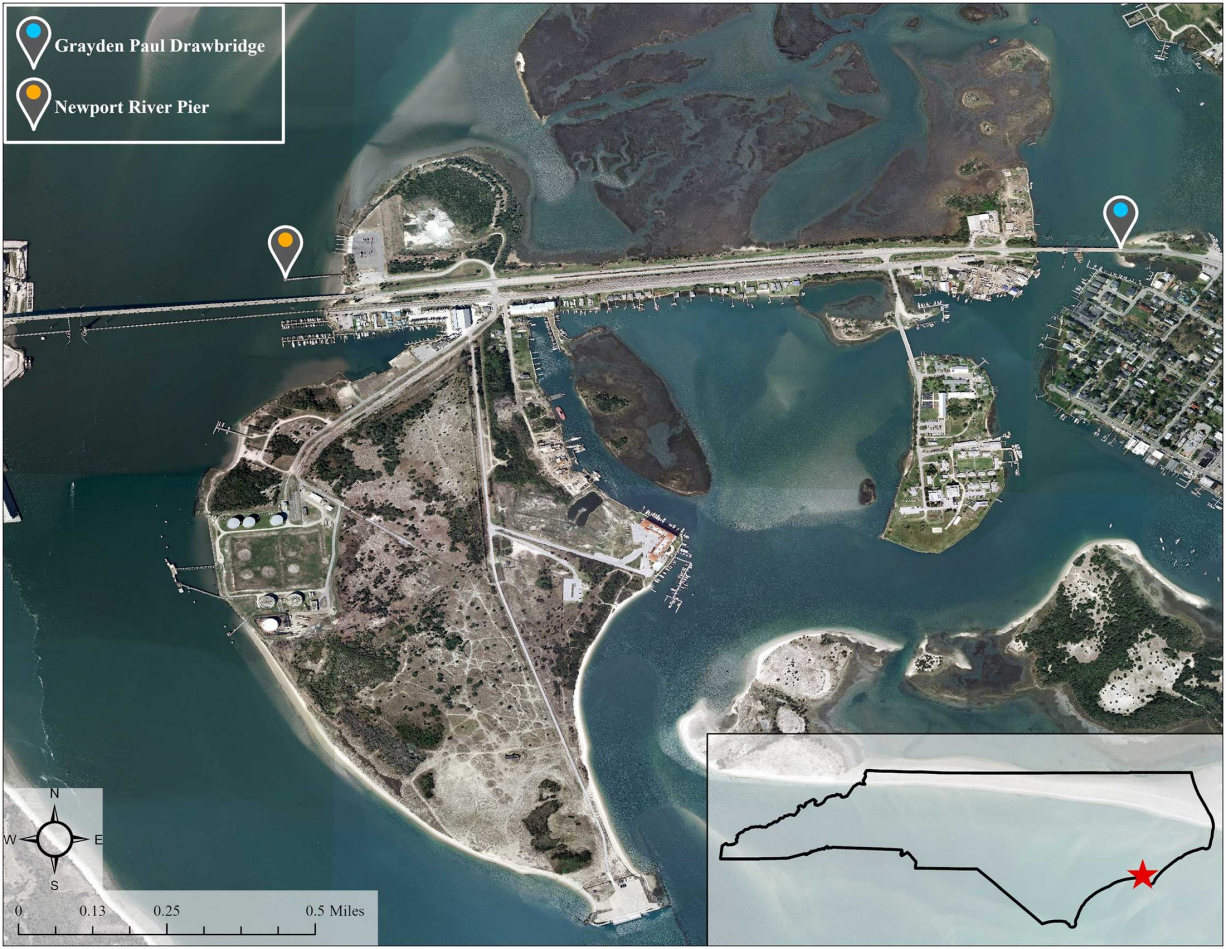
Study sites in Carteret County. Source: U.S. Geological Survey, National Geospatial Technical Operations Center, 20201211, USGS National Boundary Dataset (NBD) in North Carolina 20201211 State or Territory Shapefile: U.S. Geological Survey.

This study was approved by the Duke University IRB through permit no. 2018–0427. Oral consent to be interviewed was obtained from each participant in this study. Between June 2018 and November 2018, interviews and observations were conducted at two free fishing sites in Carteret County, North Carolina: the Newport River Pier and the Grayden Paul Drawbridge (Fig 1). The sites differ in that the Newport River Pier was built specifically for fishing and provides several amenities (lighting, fish cleaning stations, and a dedicated parking lot) while the bridge is an informal fishing site that was built for transportation and lacks amenities.

Mirroring Pitchon and Norman’s ethnographic research on subsistence communities on piers in Los Angeles, CA, this study involved semi-structured intercept interviews supplemented with more structured questions to characterize the fishing habits and demographics of anglers [13]. Some questions were also modeled on studies by Brown and Toth [14], Cooke et al. [3], and Perkinson, Faith, Vahey, Vena, and Williams [15]. Researchers asked respondents about when, where and why they fish, fishing methods, frequency of fishing, average length of fishing outing, and species targeted, as well as why they fish at the specific study locations. Fishers were asked how much of their catch they typically keep, and if they consume it or save it, and how they prepare it if they do eat it. To examine the social aspects of fishing for food, and to probe if an informal economy of sharing fish existed, fishers were asked whether they share their fish on- and off-site and who they share their catch with. They were also asked if they usually fish with anyone else, as well as if they have friends who fish in that location, or if they see the same people at each site when they fish there. To identify any information gaps on subsistence waivers and licensing, respondents were asked whether they were aware of the NCDEQ License Waivers for individuals enrolled in Food and Nutrition Services, Medicaid, or Work First Family Assistance. The NCDEQ Unified Subsistence Inland/Coastal Recreational Fishing License Waiver can be issued by the Department of Social Services (DSS) to individuals receiving benefits from the aforementioned programs.

Hurricane Florence made landfall on the North Carolina coast just miles from Carteret County on September 14, 2018, causing severe flooding and damaging both public and private infrastructure. After this event, the authors chose to incorporate questions relating to the impacts of the hurricane on fishers’ abilities to access and use fishing locations. Fishers were asked about impacts to regular fishing habits and fishing locations, number of fish caught, and perceived changes in the amount of people fishing.

Interviews were intentionally conducted at varied times throughout the day and on different days throughout the week. Everyone fishing at each study site when the researchers arrived was assigned a number based on their physical location, and a random number generator was used to select which respondents to interview. Using a “sample to saturation” strategy, interviewers collected information from multiple respondents until they were not gaining any new information [16, 17]. Interviews were recorded and quantitative responses were entered into an offline iPad survey immediately after concluding the interview.

Recorded interviews were transcribed, coded, and analyzed using QSR International NVivo 12 software. The researchers coded the interviews by categorizing the language in the responses based on themes, both those initially identified from the research questions, as well as those that emerged during the analysis. For Quality Assurance/ Quality Control purposes, and to maximize inter-coder reliability and rigor, each author independently coded an interview line-by-line while journaling general insights, complications that arose, and how they were handled. Next, the research team established guidelines regarding what type of text went into each code by comparing the individual coding structure that each researcher developed and then coding an additional interview together. This iterative coding process led to one comprehensive and rigorous coding structure that was ultimately applied to all of the interviews. The organization of language through coding allowed us to interpret the data, recognize patterns, and come to meaningful conclusions about the community we were studying.

Between the two study sites researchers approached 80 fishers and completed 74 semi-structured interviews. In the interest of time, the researchers altered the approach in the fall by concluding interviews early if respondents were not taking fish home. This applied to a total of 6 interviews which were used only to identify the overall percentage of respondents that were fishing for food, and the quantity of fish being brought home.

Fifty-four complete interviews were conducted at the Newport River Pier (32 from the summer, 22 from the fall) and 20 complete interviews were conducted at the Grayden Paul Drawbridge (7 from the summer, 13 from the fall). Interviews are cited in this paper with an “S” if conducted in summer, an “F” if conducted in the fall, an “A” if conducted at the Newport River Pier, and a “B” if conducted at the Grayden Paul bridge.

## 3. Results

### 3.1 Description of sample

Of the 80 respondents intercepted for this study, 61 respondents brought fish home for themselves or others to eat (76%). Thirty of the 39 fishers interviewed in summer (June—early August) said they bring some amount of fish home to eat, and 31 of 41 total fall (late September—mid November) fishers said they bring fish home for themselves or others to eat. Thirty-eight and a half percent of all respondents stated they would bring *all* of their catch home, 37.5% stated they would bring *some* of their catch home, and 23.8% stated they would not bring any of their catch home that day—even if they would on other occasions. Of the 32 respondents who shared how many years they have been consuming their catch, the average length was 36 years.

Demographic information on study respondents is summarized in Table 1. Of the 74 respondents that completed interviews, the majority of respondents were over the age of 40 (74.3%), white (60.8%), male (71.6%), and not Carteret County residents (77%). It is notable that the proportion of the respondents that are black or African American (35.1%) is well above Carteret County’s black or African American population (6%) [18], and also above that of neighboring Craven County (21%) [19], suggesting that more black or African American people use these fishing sites than would typically be expected given Carteret County’s racial demographics. The drivers of this trend are unknown and beyond the scope of this paper; however, it could be an interesting avenue for future research.

**Table 1:** Characteristics and demographics of fishers interviewed.

Factor	Total Sample
Gender	
n	74
% Male	71.6
% Female	28.4
Age	
n	74
% 18–24	6.8
% 25–39	18.9
% 40–60	36.5
% 61+	37.8
Race	
n	74
% White	60.8
% Black or African American	35.1
% Asian	2.7
% Hispanic or Latino	1.4
Carteret County Resident	
n	74
% Yes	23.0
% No	77.0
Distance Traveled for Non-Residents	
n	57
<1 hour	14.0
1–3 hours	40.3
3–5 hours	24.6
5+ hours	21.1
Occupation (Interview question added in Fall)	
n	34
% Employed	52.9
% Unemployed	2.9
% Retired	29.4
% On Disability	11.8
% Military	2.9

### 3.2. Social, cultural, and other perceived values of infrastructure

#### 3.2.1. Building community and sharing

Many respondents talked about the value of these fishing sites for both community and social purposes. On-site, fishing was enjoyed by family members of all ages. These publicly accessible locations were valued as a place to come with friends and meet new ones, and a place to form bonds and support others. Off-site, the reach of this public infrastructure was extended through the sharing of fish with families, with communities at fish fries, and with those in need.

At the sites many fished with family, friends, neighbors, or fishing partners. At times groups came together while others established new relationships at the sites. A new fisher to the area, for example, recognized the community at the pier and was eager to make friends while fishing, stating “I’m meeting a lot of people, talking to them” (F23A). He thought that the people at the pier were friendly and said “that’s [one of the reasons] why I moved down here” (F23A). Respondents that reported that they typically see the same people fishing stated they often became friends with them and that everyone is amicable. “Everybody I’ve met so far has been good people, they don’t lie, they don’t judge…,” offered one respondent (F9A). Among this group were older, retired fishers who often referred to people they had met while fishing as friends. Though most respondents had positive perceptions related to the social environment at these locations, other respondents said that they do not often see the same people at these locations when they fish, and do not necessarily meet or talk to others they do not know while they fish. Moreover, some mentioned occasional issues regarding people taking up too much space, fishers being rude, or themselves feeling unsafe.

Social benefits derived were not limited to socializing with others on-site. Many respondents talked about sharing their catch with others. Some fishers caught unwanted species, but instead of throwing the fish back, they would give them to others on the pier. Multiple respondents said that they would not eat mullet, for example, but one added, “If they’re close by us and if I catch a bunch of mullet, we always share the bait, and if we have too many I’m not gonna let them back in the water or let them die, so we give them to other people” (F15B). He repeatedly said that mullet is his bait, and he will not eat it, but he knows other people at the bridge do. Another respondent echoed this habit with hogfish and pinfish, which he would give away to those who wanted them because his family would not eat those specific species (F1B). Yet another respondent stated, “If I see an elderly couple who’s not doing as well as I done, I will give them some fish. Before I leave the pier. Well I mean, they’ve sat here a long time. And you notice people. And if they’re not having good luck, why not share?” (CAM 25A) This is another example that hints at social and communal concern for others on the pier. As described below, the act of sharing catch, or fishing for food for others, also extended beyond the pier.

#### 3.2.2. Fishing for food

Seventeen respondents stated that being able to catch something to eat was their main reason for fishing. This includes people who said they “like to eat fish” and those who fish “to eat,” in some cases because they rely on it. It also includes two fishers who specifically fish to obtain protein or nutrition provided by fish. Many more stated they ate their fish at different points in the interview, but did not mention eating their catch as their reason for fishing. One fisher, who became stranded in town after Hurricane Florence, represented the truest form of subsistence encountered by the researchers. He stated, “We’re not sports fishermen, we fish to eat. What we catch, we grill… We fish for food. The missions don’t always give you anything good to eat, they give you peanut butter and crackers and bread. I like meat” (F9A). He continued on, “See that black SUV by the garbage can? That’s our home. I’m not paying $120/night for some regular roach motel…” When asked how often they fish, he stated, “Regularly, like 3 times per week, but now it’s like every day just to survive” (F9A).

Some respondents reported fishing specifically for the nutritional benefits that fish provide as a healthy source of protein. One senior interviewed at the Grayden Paul Drawbridge explained, “I like the taste and it’s good… and it is healthy. Healthiest meat I eat… is fish” (S1B). Explaining another health benefit, another respondent stated that he needs the fish to meet protein requirements dictated by his doctor for his diabetes, saying, “Fish, fish have a lot of protein and that’s what they tell me I need” (F7B). The ability to catch his own fish provides him a cheap, accessible way to meet his nutritional needs.

For others, fishing for food is about providing for friends and family that rely on it, highlighting an informal economy that exists both on and off public infrastructure. After leaving the fishing sites, many respondents described family members, non-relatives residing in their households, and simply those present when they brought home their catch as the people they most often share their fish with. The amount shared was often based on how much was caught, and respondents said they typically shared with anywhere from a few family members to several dozen members of their social networks or religious communities. Indeed, the sharing beyond the pier and beyond immediate family members could sometimes be quite extensive. For example, one respondent (local to Carteret County) described catching 60 fish at a time to feed four families per week:

We help feed the people in North River [a community about ten miles away from our study sites]… Like we’ll take ’em all the fish. I donate most of the fish I catch… There’s a lot of people from where I’m from that I know need help, so. I know they love fish too. A couple of my friends’ moms who can’t work, they’re disabled and stuff like that. I’ll clean fish and take it to ’em or just take ’em a Ziploc bag slammed full of fish (S3B).

Another respondent reported fishing to feed older neighbors and friends, saying, “they pretty much rely on me… I’ve got enough people down there—older people—who I supply with fish and stuff, too… When they come to me, they need fish, you know?” (F11B). Children were also frequent beneficiaries: twenty-five respondents said they shared their catch with children. Ten of these respondents explicitly stated how many kids they shared their catch with, averaging two children per fisher. A retiree from a county further inland pointed to the steep price of fish as a reason why others rely on the fish he catches (F16A). However, not all those who shared their catch with family and friends shared it because others depended on it—some shared their catch simply because others enjoyed it. One young man at the Newport River Pier who shared his catch stated, “I know they enjoy it themselves. I mean they’re not starving if I don’t get it” (F11A).

Other respondents described fishing habits that hinted at a level of food dependence but did not explicitly describe it as such. For example, one woman said that she fishes for nine to fourteen days until her freezer is filled, then takes a week off and comes back to fish for another nine to fourteen days. Similar to a lot of respondents, she has been fishing frequently for decades and keeps her catch, but does not describe it as an important part of her diet (F25A). In one instance, an angler spoke of bartering with his fish, trading his catch for beans, or other agricultural products. Others traded different species of fish, and more skilled (or lucky) fishers gave their fish away to those on-site they deemed might need it most (i.e., older people or those who “look like they are fishing for food” (S2A)).

#### 3.2.3. Recreation and mental health

In addition to the community building and consumption motivations described above (which were described by the majority), respondents shared other motivations for fishing, listed in Table 2. Over one third of respondents mentioned they fish for recreation, stating they fished for “fun,” or “enjoyment,” or simply because they “like it.” Some respondents explained that they fish because they like the “sport” or competition associated with fishing. Relaxation and mental health benefits were among other common reasons interview subjects stated they fish, including stress relief, socialization, and other avenues of supporting their emotional health. “This is pretty much therapy. I come down, I catch a fish, I feel,” stated one fisher (F21A), and “it’s my mental down,” stated another (S2A). Another offered that fishing was “just relaxing. I’ve got all the time in the world. Retired, you know” (F23A). Others mentioned that fishing was an important part of their lifestyle or family tradition. “I used to go with my granddaddy and my daddy, you know, 60 years” offered one respondent, and “I was raised fishing…I started at 3-years-old, and I’ve been fishing ever since and I’m 66 now” said another (F14A, F25A).

**Table 2:** Fishers’ responses to the question, “what is the main reason you fish?” if a respondent mentioned more than one reason for fishing in response to this particular question, they were included in each category.

REASONS FOR FISHING	
**FUN**	27
**FOOD**	17
**RELAXATION & MENTAL HEALTH**	16
**LIFESTYLE AND TRADITION**	9
**SPORT**	8

#### 3.2.4. Physical accessibility

Nine fishers noted that they benefited from the physical accessibility provided by public infrastructure, including the safety it provided them while fishing as well as convenience. Fall interviews included a question about fishers’ occupations. Of the 35 individuals that offered a response to this question, ten stated they were retired (29%), and another four noted they were “on disability” (11%). Those who were not working due to retirement or a disability often fished as a way to fill their time. One disabled respondent described their appreciation for having a place to fish where others were frequently around, due to safety concerns (F7B).

A number of other respondents recognized the utility of these fishing locations for those who were physically disabled. A man that frequently fished at the Grayden Paul bridge appreciated the accessibility of the bridge for his sister who uses a wheelchair. When she arrived at the bridge, she benefited by being able to drive all the way up to her brother, before getting out of the car and into her wheelchair (F6B). Another respondent also mentioned the accessibility of the Newport River Pier as a major draw for why she fishes there with her nephew who uses a wheelchair (S2A). We observed two other men fishing from wheelchairs at different points in the summer.

The bridge and pier also provide physically convenient and affordable places to fish. When fishers were asked why they fish at these locations, one common response was that fish like structure and that the pier and the bridge were good fishing sites because of the structure they provide. People also appreciated that infrastructure increases their access to fish by allowing them to fish into deeper, otherwise inaccessible areas. Multiple respondents also noted the comfort and ease of piers, and being able to avoid getting sandy (particularly compared to beach/surf fishing, which is also freely accessible). Fishers frequented both sites because of their convenience and proximity to home. At both locations, fishers talked about public infrastructure as an alternative to fishing off of a boat, either because of weather conditions rendering it unsafe or unsuitable for fishing, or because they could not afford a boat or did not have access to one.

Respondents commonly noted that both sites are free places to fish, making them more appealing than other pay-to-fish piers in the area. “I’m a staunch advocate of public access… You know, as the very wealthy buy up properties and do all that… it’s really important to have it [public access] so that the ordinary person can come down here,” stated one respondent (S9A). Another fisher interviewed at the pier noted, “I’ve noticed they’ve put in a lot more boat ramps, they’ve improved all the ramps, put in fishing piers everywhere. They didn’t last long because of the hurricane, but they put them in… I’ve noticed a lot of improvements” (F13A).

#### 3.2.5. Hurricane Florence’s effect on access

Almost 70% of the fishers interviewed after Hurricane Florence described their fishing activities as negatively affected by the hurricane in some way. Some respondents mentioned an inability to fish as frequently as a result of being displaced or needing to tend to repairs, while others perceived lower catch levels since the storm. The most notable impact, however, was structural damage to popular fishing sites. More than half of the respondents interviewed post-hurricane described damage to places they commonly fish. A majority described damage to piers, though a few mentioned impacts to small, local fishing spots such as bridges or shore-based locations.

Hurricane impacts on fishing locations affected fishers differently. For example, the respondent who fishes nine to fourteen days at a time every other week (F25A) was likely more severely affected by damage to the sites at which she fished than someone who fished less frequently. Similarly, the people who were most affected by the hurricane were those whose preferred fishing sites were damaged. While a site may have sustained damage, it does not necessarily imply that the site had lost all of its fishing value. For example, one woman said about Oceanana Pier (another local pier fishing location), “Well, yeah, it did tear off the end of the pier, but usually we didn’t fish on the end” (F24A).

### 3.3. NCDEQ subsistence license waivers

Information about the subsistence fishing license waivers that are provided to individuals enrolled in Food and Nutrition Services, Medicaid, or Work First Family Assistance programs is only provided upon request. At the time of data gathering, information about this waiver was only publicly advertised in the annual regulations digest [20], and the Department of Social Services was “not required to disclaim” any of their services including the waivers as revealed in a phone call between researchers and the department in October 2018. A public records request revealed that 750 subsistence license waivers were granted in Carteret County in the 2017–2018 fiscal year, and 966 in neighboring Craven County [21]. This represents 9.2% of the 8,125 total recreational fishing licenses granted in Carteret County for the year, and 13.7% of the 7,043 in Craven County.

When asked if they were aware that NCDEQ offers free fishing licenses to those who qualify, respondents occasionally thought that this question referred to lifetime licenses or reduced-cost licenses for seniors. As of 2018, NCDEQ offered senior lifetime licenses for $15 for residents over 65. In general, people were more likely to know about the senior lifetime program than the subsistence waivers, perhaps because of the relatively high percentage of respondents ages 61 and over (38%). Overall, the people who knew about the subsistence waivers tended to have one themselves or know someone who had one. Those who knew about the waivers, but not through personal experience, also tended to have heard through word of mouth, rather than seeing something posted.

Generally, people did not know about these subsistence license waivers but were of the opinion that they would be useful to others who fished at the Newport River Pier and Grayden Paul Drawbridge. Multiple respondents at both the bridge and the pier mentioned the usefulness of the waivers specific to this particular locale. “I mean, this pier in particular draws a lot of local folks whose resources are limited. So yeah, I absolutely believe it would be beneficial” shared one fisher (F12A). Another fisher, who said he was fishing for “survival,” stated, “Y’all need to put a sign up with that right there. Most of the people down here come fish to get something to eat. Most of them are retired” (F9A). A Carteret County resident, with firsthand knowledge of the area, concurred, saying that, “there’s a lot of people that fish down here because they need to supplement their food stores in their house. So if they knew they were able to get a free fishing license… more people would probably be out here” (F21A). Another respondent cited the usefulness of these waivers, saying, “Yeah, because I was here one time and the game warden came out here and cleared the deck… he came out and started asking for fishing licenses and I would say 90 percent of people in the pier didn’t have a license, and they were older people” (F20A). A majority of respondents that answered this question thought that the information about these waivers was not widely or effectively distributed. Multiple respondents expressed sentiments about the lack of availability of information on the waivers. One elaborated that “unless you went looking for it, you wouldn’t know where to find it” (F6B). Only two respondents expressed negative views on the NCDEQ fishing license subsistence waivers, citing that they felt it was unfair for some to have to pay for a license while others do not.

## 4. Discussion

This study contributes to the sparse bodies of literature on the social, economic, and cultural characteristics of individuals who fish for food in the United States and how public infrastructure facilitates access to nutritional, social, recreational, cultural, and mental health benefits associated with fishing for food. This exploratory study addresses that gap and begins to characterize a community of recreational fishers in North Carolina who consume their catch and how they, and their social networks, benefit from this activity. Results show a wide range of benefits that public fishing infrastructure facilitates for residents of Carteret County and, likely by extension through the sharing of caught fish, elsewhere in the state. Outside of just North Carolina, according to the US Fish and Wildlife Service’s 2016 National Survey of Fishing, Hunting, and Wildlife-Associated Recreation, there were more than 7 million recreational fishers over the age of 16 in the South Atlantic region, more than in any other national census region [22]. Though our research is local to Carteret County, it is likely that a contingent of those 7 million fishers are using similar fishing infrastructure and gaining similar benefits to those found here. While many respondents acknowledged that these sites also support people who are truly fishing for subsistence, we have shown how these fishing sites are meaningful in ways beyond just subsistence. This involves physically accessible places that can provide healthy food, access to recreation, a free family activity and a way to carry on a family tradition, a way to improve mental health, and a place to bring and meet friends. These sites also assume a critical role in supporting informal community economies, characterized by sharing fish on- and off-site. Reliable access to the values and benefits provided by these sites is in part mediated by knowledge of licensing regulations, in particular subsistence license waivers.

### 4.1. Fishing for food as a socio-cultural activity

#### 4.1.1. Informal economy as an emerging theme

The sharing of fish for both bait and consumption constitutes an informal economy of sharing, characterized by different forms of exchanges (giving vs. trading) as well as a number of different incentives for sharing (e.g. enhancing social ties, providing for those in need, sharing with those who simply enjoy consuming fish). Many of those interviewed derived or provided social and economic benefits stemming from their ability to access these fishing locations and share their catch. For those who fished to provide for community events, like fish fries and religious gatherings, the sharing of fish supported a cultural activity. Fishers gave their catch to others on-site, shared with friends, family, and neighbors off-site, and in some cases fed entire families in their communities. Some even traded for other fish or agricultural products. This sharing and trading of fish represents an informal economy both on and off of public infrastructure, within existing social circles, and even among strangers who encounter each other at formal and informal fishing sites.

This concept of an informal economy of sharing fish and the social benefits it supports is not unique to our study. In Tyrrell County, NC, Brown-Pickren and Manda found that 66% of fishers fish to help feed their families [5]. In describing this informal economy, they found that it is not solely based on need, but also enjoyment and the enhancement of social ties, echoing Steinback et al., who found that the widespread sharing of fish extended the benefits of fishing by both those fishing for recreation and those explicitly fishing for supplementary food or income [4]. As part of her research on fishing for food in urbanized Connecticut, Ebbin similarly explored this informal economy of sharing and trading fish, and found that those fishing and harvesting food attached significant social and cultural values to this activity [6], echoing the important role that resource sharing plays in supporting social connections found by Pulford et al. [7]. Respondents in our study explicitly highlighted the value of formal and informal fishing infrastructure for the purpose of supporting community and social ties. The sharing of fish for both bait and consumption extends the positive benefits (nutritional, social, etc.) of public fishing infrastructure to even more individuals, acting as part of an informal, but important economy.

#### 4.1.2. Revisiting traditional characterizations of fishing

The Grayden Paul Drawbridge and Newport River Pier seem to contribute significantly to fishers feeding themselves, an outcome outside of the typical narrative that recreational fishing occurs primarily for enjoyment. The benefits associated with an informal economy in these results also highlight the insufficiency of strict definitions of “subsistence” to capture either the motivations of, or the benefits to, those who fish for food. In her examination of how commercial and recreational fishermen might construct ideas of fishing, Boucquey suggests “commercial fishermen rely on fish for a livelihood and to cement community relationships”; whereas, recreational fishermen have a more individualized relationship to fish arising from notions of fun, game fishing, winning a prize, and a sense of purpose [23]. Fishing for food does not fit neatly into either of these categories. The mix of sharing (informal economy), level of dependence, and enjoyment suggest that this type of fishing is a hybrid category exhibiting elements of all three (commercial, recreational, and subsistence) and that the free, public venues (the study sites) provide accessible spaces that generate benefits not accounted for in standard fisheries data.

### 4.2. Fishing infrastructure as a means to access benefits

#### 4.2.1. Fishing for food

While a variety of motivations exist for this activity, it is clear that some people fish for food that they, or others, rely on for some portion of their diet. While some did not describe being ‘reliant’ on the fish they catch, the frequency of fishing and retaining catch, and length of time spent fishing for food, suggest that this activity provides a number of meals (benefits) for them. These findings are similar to those highlighted in the Ebbin study in Connecticut, where despite the nearly 43% of respondents that cited fishing for food as an important motivation for fishing, most of those approached did not consider themselves to fall into the category of ‘subsistence’ fishing—a term which nearly half of the fishers in that study were aware of [6]. While reasons for an apparent reluctance to identify as a ‘subsistence fisher’ are unclear, it could be due to prevalent societal notions that subsistence activities are associated with low-income, impoverished communities. Respondents that weren’t necessarily fishing for their *own* food often fished to provide their families and social network with food, in some cases noting that these individuals relied on the catch. The access to food provided through fishing might be particularly important to certain individuals, such as those who are retired or on disability with limited income.

#### 4.2.2. Recreation and mental health

In addition to food-provisioning benefits associated with fishing for food, our respondents also cited well-being benefits, like enjoyment, mental health, and socialization. Respondents described fishing for fun or enjoyment, which was also the most popularly-cited reason for fishing in Ebbin’s study [6]. A portion of our respondents stated that they fish because of the “therapy,” “mental down-time,” relaxation, or stress-relief benefits, echoing Ebbin’s study which found relaxation to be the third most popular reason for fishing [6]. In a study in Western Australia examining the health and well-being benefits gained from recreational fishing, 75% of respondents found fishing provided them with a “positive state of mind,” using words like “mental health,” “stress relief,” and “clarity of mind” to describe this benefit [24]. That study concluded that these benefits warrant the promotion of recreational fishing, a conclusion we echo.

#### 4.2.3. Physical accessibility

The physical accessibility of both study sites was a clear benefit to many users and many different types of people, including those who were elderly or disabled, were able to utilize the infrastructure. The sizable elderly and retired population at the study sites benefited from the more protected environment and flat surfaces offered by the pier and the bridge, as compared to shore-based or boat-based fishing. This infrastructure offers an alternative to walking on uneven sand, or to embarking and disembarking from a boat, making piers and bridges particularly important for fishers with disabilities and mobility challenges.

Fishing access for people with disabilities is not a heavily studied area, and most existing literature points to how formal fishing sites can be retrofitted to improve this access. While accessibility is a requirement (under the Americans with Disabilities Act) at formal fishing piers and platforms and at boating facilities, it does not apply to informal fishing sites sometimes used by people fishing for food [25]. Because this invisible activity can occur off of infrastructure not constructed for the purpose of fishing, there may not be any legal requirements in place to ensure accessibility for people with disabilities. In our study, the Grayden Paul Drawbridge, although not subject to ADA requirements, was valued for its accessibility and was both utilized and appreciated by people with mobility challenges. This suggests the importance of considering and preserving informal infrastructure that provides specialized access values for older fishers and people with disabilities, values that wouldn’t typically be considered due to the informal nature of the infrastructure.

#### 4.2.4. Hurricane Florence’s effect on access to benefits

The impacts of Hurricane Florence on popular fishing sites provided some insights into how fishers might be affected if these sites are not maintained. Hurricane Florence impacted people’s frequency of fishing and ability to reap the benefits associated with access to formal and informal fishing infrastructure. It also could have limited the ability for some of those fishing for food to access their typical catch, reducing overall food security. Similarly, Brown-Pickren and Manda found that frequent flooding impacted access to fishing in Tyrrell County, NC, acting as a barrier to access through the drowning or washing-away of fishing spots [5]. While Brown-Pickren and Manda were not focused on physical fishing infrastructure, these natural events and disasters obstructed access to fishing and its associated benefits by limiting access to fishing sites as a result of damage [5]. In addition to suggesting a need for regular maintenance of fishing sites, these examples point to the importance of prioritizing repairs to fishing sites and infrastructure, whether formal or informal, after destructive natural events.

These sites face more threats than just natural disasters. By the time of final data collection efforts at the Grayden Paul Drawbridge in November 2018, access to the bridge was blocked and local traffic had been rerouted over a nearby new bridge, which is much larger and not suitable for fishing. Due to the informal nature of this fishing location, it is unclear whether fishers’ access values were considered in the decision to tear down the bridge. The area has since been turned into a park, and while fishers can still be seen fishing along the seawall, it is unclear whether fishers derive the same benefits they previously derived.

### 4.3. Knowledge of NCDEQ subsistence license waivers

A significant finding of this study was the lack of awareness about NCDEQ subsistence license waivers among the interviewed population. There was limited knowledge of this waiver program, yet widespread acknowledgement of its potential usefulness. This finding echoes that of Brown-Pickren and Manda in Tyrrell County, NC, where they found subsistence fishing waivers to be beneficial to the community, yet underutilized [5]. Our study revealed a population reliant (to varying degrees) on the fish caught at the study sites, whether directly or indirectly. Respondents also recalled instances where people utilized the two study sites without fishing licenses. Comparatively, the study in Tyrrell County found that roughly half of respondents interviewed were aware of the NCDEQ subsistence fishing license waivers, though nearly a quarter still fished without a license [5]. The authors suggest loosening restrictions on attaining the subsistence waivers to help increase secure access to fishing [5].

Our findings also suggest an opportunity to increase access to licenses for fishermen. Better advertising of the subsistence license waivers could help fishers avoid significant fines associated with being caught without a fishing license, avoid burdens on lower-income fishers, and enable the state government to account for recreational fishermen that might not otherwise be licensed. Given concerns about the contributions of recreational fishing to bycatch and overfishing [23, 26–30], promoting and extending access to these waivers could be valuable in providing more accurate catch and licensing statistics, resulting in improved overall accuracy of state fishery statistics. In addition to the importance of accurate data for resource management purposes, the number of licensed anglers in a state affects funding allocations for state fish and wildlife agencies, resulting in financial consequences for states in which fishers are undercounted [31]. Additionally, Ebbin notes that while the harvesting of marine resources may not always be visible, a better understanding of subsistence and personal consumption by harvesters and their families could aid regulators in identifying regulatory shortfalls and guide them to more appropriate policies meeting the needs of those fishing for food [6].

A potential step forward could be to enhance NCDEQ’s partnership with county Departments of Social Services to jointly promote the waivers, perhaps at places frequented by recreational fishers (e.g. bait shops, popular fishing spots). A similar strategy could include making the subsistence fishing license waivers a standard talking point for case workers at DSS when consulting with citizens on SNAP (Supplemental Nutrition Assistance Program). It is worth noting that, prior to this study, these waivers were only mentioned by the Department of Social Services after a specific inquiry. After sharing our results with the Carteret County DSS, the agency sent out a press release urging all Health Department and DSS staff, the Consolidated Human Services Board, and 18 media contacts to “remind residents who receive Medicaid, Food Stamps, or Work First Family Assistance [that] they qualify for a free twelve-month fishing licensing waiver” (personal communication with Cindy Holman, 7/15/2019). DSS also mentioned that they were researching the possibility of providing staff with a flyer or brochure that they could distribute about the waivers when taking applications from people attempting to enroll in these programs (personal communication with Cindy Holman, 6/6/2019). We hope that efforts like these will increase awareness and uptake of these waivers.

## 5. Conclusion

This study provides insights into a largely understudied fishing population, documenting a variety of benefits that people derive from fishing for food, including fun, nutrition, sport, mental health, and tradition. It also illustrates the multiple values of public fishing infrastructure like the pier and bridge and suggests that the benefits fishers (and their networks) enjoy through access to public fishing infrastructure are compelling rationales for the creation and maintenance of both formal and informal fishing places. The findings reveal that a considerable population of those fishing in the study locations depend on that infrastructure to fish for food, and that a portion of them, their families, and their communities, rely on the access to this food source. This has implications for food security and is central to an informal economy of sharing. Many respondents would also likely benefit from continued access to infrastructure, access to licenses, and information about those licenses. Future studies could assess if these values and benefits differ from those on paid piers rather than free public infrastructure, the patterns of fishing for food and dependence along racial or gender lines, or explore similar questions with structured quantitative surveys, allowing researchers to quantify catch volume and socioeconomic status of this group. Overall, we intend for this study to draw attention to this understudied population of recreational fishers and make them visible in management decision-making. In uncovering the range of values which fishermen attribute to infrastructure, we begin to make the case that public fishing spaces, both formal and informal, are worth the federal and state expenses required to upkeep and preserve these sites.

## Supporting information

S1 Table Seasonal species targets identified by respondents. Respondents were asked what species of fish they target in each season. The number of people that mentioned each species target in each season are identified in S1 Table. Summer interviews only asked about summer targets. In the fall interviews, follow-up questions asked about species targeted in the fall, spring, and winter.
(PDF)
