# Supplementary material for: Fishing for food: Values and benefits associated with coastal infrastructure
Source: PLoS One. 2021 Apr 15;16(4):e0249725. doi: 10.1371/journal.pone.0249725 (PMC8049240; doi:10.1371/journal.pone.0249725)
Supplement: S1 Table — Respondents were asked what species of fish they target in each season. The number of people that mentioned each species target in each season are identified in S1 Table. Summer interviews only asked about summer targets. In the fall interviews, follow-up questions asked about species targeted in the fall, spring, and winter. (PDF) [file pone.0249725.s001.pdf]

S1 Table. Seasonal species targets identified by respondents.

| | Summer | Fall | Spring | Winter | Total |
|---|---|---|---|---|---|
| Anything | 16 | 2 | 1 | 2 | 21 |
| Bluefish | 8 | 2 | 1 | 2 | 13 |
| Red Drum | 9 | 6 | 4 | 3 | 22 |
| Black Drum | 3 | 1 | 1 | 1 | 6 |
| Drum | 5 | 3 | 1 | 0 | 9 |
| Puppy Drum | 1 | 0 | 0 | 1 | 2 |
| Speckled Trout | 1 | 2 | 2 | 2 | 7 |
| Trout | 8 | 9 | 1 | 4 | 22 |
| Flounder | 19 | 7 | 5 | 5 | 36 |
| Spot | 12 | 13 | 4 | 9 | 38 |
| Sea Bass | 2 | 1 | 1 | 1 | 5 |
| Croaker | 7 | 2 | 0 | 2 | 11 |
| Sea Mullet | 7 | 3 | 2 | 3 | 15 |
| Hogfish | 3 | 5 | 1 | 2 | 11 |
| Pufferfish | 4 | 3 | 1 | 2 | 10 |
| Pinfish | 2 | 2 | 1 | 1 | 6 |
| Spanish Mackerel | 1 | 0 | 0 | 0 | 7 |
| King Mackerel | 1 | 0 | 0 | 0 | 1 |
| Whiting | 1 | 2 | 1 | 1 | 5 |
| Catfish | 0 | 1 | 0 | 0 | 1 |
| Grouper | 1 | 1 | 1 | 1 | 4 |
| Sea Robins | 1 | 0 | 0 | 0 | 1 |
| Perch | 1 | 0 | 0 | 0 | 1 |

| | | | | | |
|---|---|---|---|---|---|
| Crappie | 0 | 0 | 0 | 2 | 2 |
| Snapper | 0 | 0 | 1 | 1 | 2 |
| Sheepshead | 3 | 0 | 0 | 0 | 3 |
| Sharks | 3 | 0 | 0 | 1 | 4 |
| Crabs | 1 | 0 | 0 | 0 | 1 |
